# Wearable Fetal ECG Monitoring System from Abdominal Electrocardiography Recording

**DOI:** 10.3390/bios12070475

**Published:** 2022-06-30

**Authors:** Yuwei Zhang, Aihua Gu, Zhijun Xiao, Yantao Xing, Chenxi Yang, Jianqing Li, Chengyu Liu

**Affiliations:** 1 The State Key Laboratory of Bioelectronics, School of Biological Sciences and Medical Engineering, Southeast University, Nanjing 210096, China; zhangyuwei@seu.edu.cn; 2 The State Key Laboratory of Bioelectronics, School of Instrument Science and Engineering, Southeast University, Nanjing 210096, China; zhijunxiao@seu.edu.cn (Z.X.); 230198304@seu.edu.cn (Y.X.); chenxiyang@seu.edu.cn (C.Y.); ljq@seu.edu.cn (J.L.); 3 State Key Laboratory of Reproductive Medicine, Institute of Toxicology, School of Public Health, Nanjing Medical University, Nanjing 211166, China

**Keywords:** fetal, electrocardiography (ECG), health status, monitoring system, fetal heart rate (FHR)

## Abstract

Fetal electrocardiography (ECG) monitoring during pregnancy can provide crucial information for assessing the fetus’s health status and making timely decisions. This paper proposes a portable ECG monitoring system to record the abdominal ECG (AECG) of the pregnant woman, comprising both maternal ECG (MECG) and fetal ECG (FECG), which could be applied to fetal heart rate (FHR) monitoring at the home setting. The ECG monitoring system is based on data acquisition circuits, data transmission module, and signal analysis platform, which consists of low input-referred noise, high input impedance, and high resolution. The combination of the adaptive dual threshold (ADT) and the independent component analysis (ICA) algorithm is employed to extract the FECG from the AECG signals. To validate the performance of the proposed system, AECG is recorded and analyzed of pregnant women in three different postures (supine, seated, and standing). The result shows that the proposed system can record the AECG in different postures with good signal quality and high accuracy in fetal ECG and heart rate information. Sensitivity (*Se*), positive predictive accuracy (*PPV*), accuracy (*ACC*), and their harmonic mean (*F*1) are utilized as the metrics to evaluate the performance of the fetal QRS (fQRS) complexes extraction. The average Se, PPV, ACC, and F1 score are 99.62%, 97.90%, 97.40%, and 98.66% for the fQRS complexes extraction,, respectively. This paper shows the proposed system has a promising application in fetal health monitoring.

## 1. Introduction

Perinatal complications contribute to approximately 40% of the total perinatal and maternal deaths, whereas heart defect is an important factor in perinatal stillbirth worldwide [1]. Therefore, monitoring the status of the fetal heart rate (FHR) is of paramount importance during pregnancy or delivery [2].

Currently, as the gold standard for FHR monitoring, cardiotocography (CTG) provides a visual representation of FHR and uterine contractions. Nevertheless, CTG only provides an estimate of the FHR and is prone to signal loss and cannot be used for a long time. Doppler ultrasound is routinely employed during pregnancy and delivery [3]. However, ultrasound is not passive and requires frequent repositioning of the ultrasound transducer. Thus, to monitor the fetus’s health during daily life, a comfortable, safe, long-term fetal monitoring system that can be used conveniently is needed.

Studies reveal that a fetal electrocardiogram (FECG) estimates the fetal heart movement, which has the potential to provide rhythm information and morphology, such as the PR and QT intervals or ST segments [4]. FECG monitoring is a convenient scheme for early detection and diagnosis of fetal congenital heart disease and distress [5]. In practice, the FECG signals can be collected in two strategies: fetal scalp electrocardiography (SECG) and maternal abdomen electrocardiography (AECG). SECG is capable of providing accurate FECG morphology and fetal heart rate (FHR). Nevertheless, SECG is invasive, expensive, and requires skilled personnel. In contrast, AECG is inexpensive, convenient, and has no harm to the mother and the fetus during the pregnancy. Additionally, AECG can work earlier weeks (>20 weeks) while SECG monitors the fetus’s health during labor exclusively. Therefore, developing a non-invasive FECG (NI-FECG) monitoring system is essential for early heart disease detection, which can improve the effectiveness of appropriate treatment for the fetus. At the present, non-invasive physiological measurement has gradually become a new trend [6].

Currently, several devices are available that can acquire FECG signals [7,8,9,10,11,12,13,14,15], such as Avalon (with wireless transducer) [7] and AN24 [8]. In addition, Fanelli et al. [10] developed remote fetal care to monitor fetal health during pregnancy. The monitoring system could help pregnant women monitor the status of themselves and their fetuses at home. Le et al. [11] designed a home-based mobile maternal and fetal ECG acquisition, which includes a cloud assistant. Yuan et al. [12] established a fetal ECG monitoring system based on the android smartphone. Galli et al. [13] developed a fetal heart rate monitoring using multiple dry electrodes.

There are two main technology challenges in implementing a home-based NI-FECG monitoring system. The first challenge comes from the feasible hardware acquisition module of the wearable FECG monitoring system that ensures the maternal AECG signal is continuously collected in different states. Secondly, accurate and real-time waveform analysis requires professional knowledge to be properly employed to the AECG signal of the pregnant woman.

Wearable ECG monitoring is produced by the traditional ECG detection fusion materials, electronics, information, artificial intelligence, and other emerging technologies, which is increasingly prevalent in the push toward autonomous health monitoring [16,17,18,19,20]. It can be worn by pregnant women to obtain the AECG signals and to realize continuous and long-term dynamic monitoring of pregnant women. The collected maternal AECG signals incorporate lots of noise interference such as baseline drift, power line, electromyography (EMG), mother’s breathing interference, and motion artifacts [21]. In particular, the amplitude of the maternal ECG (MECG) is often several times that of the FECG in the ECG signal detected from the abdomen, which makes the extraction of the FECG quite difficult [22].

Although significant progress has been made in ECG signal processing in the past few years, the analysis of the FECG signal is still in the early stages of development. Several works of literature were presented on the location of the fetal QRS (fQRS) complexes utilizing the AECG recordings [23]. The methods proposed in the literature include convolutional neural network (CNN) [24], template subtraction (TS) [25], the least mean square (LMS) adaptive filter (AF), the recursive least square (RLS) adaptive filter [26], Kalman filtering (KF) [27], independent component analysis (ICA) [28], periodic component analysis (πCA) [29], principal component analysis (PCA), wavelet transform (WT), the echo state neural network (ESN), and fusion of different methods (FUSE method) [30], etc.

This paper aims to design a portable, home-based, FECG monitoring system, which can be used for continuous monitoring of fetal health. The contributions of this work are as follows.

1. Considering that fetal ECG is very weak and vulnerable to noise, a high-precision, low-noise portable ECG measure device is designed and optimized to collect pregnant women’s abdominal ECG signals in different states (supine, seated, and standing posture). The system consists of biocompatible electrode materials, noise suppression design and amplification circuit, data transmission, and storage module;
2. A major prerequisite in non-invasive AECG recordings analysis is the accurate extraction of FECG signals in the presence of background noise and maternal artifacts. We present an effective algorithm for AECG signal analysis, including signal pre-processing, maternal QRS location, maternal ECG subtraction, and fetal QRS complex detection.

This paper is organized as follows. Section 2 presents the design of the FECG monitoring system briefly. Section 3 illustrates the algorithm for signal analysis. Section 4 details the experiment designs and results. Section 5 demonstrates the discussion. Finally, the conclusion is drawn.

## 2. Design of FECG Monitoring System

**Figure 1:**
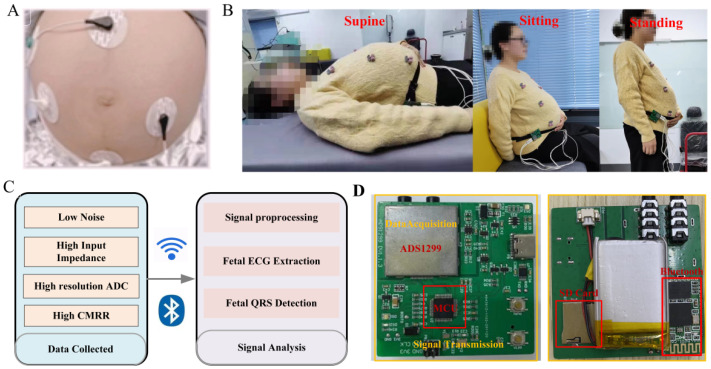
The diagram of the fetal monitoring system frame. (**A**) Electrode placement. (**B**) Postures of the subject. (**C**) Monitoring system. (**D**) Hardware prototype.

The diagram of the fetal monitoring system frame is presented in Figure 1. The monitoring system mainly includes a data acquisition module, data transmission module, signal storage module, and signal analysis platform. Electrodes will be attached to the skin in a certain way for the collection of pregnant women’s AECG signals. The signal acquisition module filters and amplifies the AECG signals (including maternal ECG signals and fetal ECG signals) and converts them from an analog signal to a digital signal. The signal transmission module transmits the abdominal ECG signals to the PC interface by Bluetooth or stores them in a memory card. The signal analysis platform displays the AECG waveform and processes and analyzes the AECG data.

### 2.1. Electrode

The electrode is an important medium for connecting pregnant women’s abdomen and signal conditioning circuits. Its performance directly affects the quality of the collected signals and the comfort and reliability of the pregnant women during the use of the equipment. To accurately and effectively collect the fetal ECG signal and ensure that the signal has a high signal-to-noise ratio (SNR), the electrode demands meet specific performance indicators. The AgCl electrode has good conductivity, low noise, and a stable baseline, which can ensure high-quality signal acquisition.

It is investigated that three linearly independent ECG electrodes can be used to construct a surface ECG vector map [31]. The electrode placement is designed with three acquisition channels, a reference point, and a left leg drive. Determine the reference electrode point 5 cm below the center of the pregnant woman’s navel. Three acquisition electrodes draw a triangle around the navel. The left leg drive electrode is on the right side of the participant. This configuration is chosen for the reason that it maximizes the SNR [32].

### 2.2. Signal Acquisition Module

When recording fetal ECG detection, instantaneous high voltage may be generated due to the influence of the external environment, causing damage to the entire hardware circuit. In addition, a lot of electromagnetic interference exists in the working environment of ECG acquisition equipment. A buffer is designed to increase input impedance and improve load capacity and noise immunity. Simultaneously, a preprocessing circuit is added to the hardware acquisition system composed of a second-order passive low-pass filter and a limiting circuit, which plays a crucial role in eliminating high-frequency interference and overvoltage protection.

The fetal ECG signal is relatively weak, and the amplitude of the fetal ECG is varied from 10 µV to 60 µV. It is susceptible to maternal interference, myoelectric interference, and power frequency interference [33]. Therefore, the hardware acquisition system is required to have low noise, high input impedance, and a high common-mode rejection ratio (CMRR). The data acquisition module of this system is implemented with an analog front end ADS1299 (Texas Instruments, Dallas, TX, USA), which includes a programmable gain amplifier (PGA), high-precision analog to digital converter (ADC), and right leg drive (RLD). Input referred noise, input impedance, and CMRR of the ADS1299 are 1 μVpp, 1000 MΩ, and −110 dB, respectively, which meets the requirements of the acquisition system.

The AECG acquisition process is often subject to common-mode interference introduced by the power line or other interference sources. The RLD circuit detects the common-mode component in the input signal and feeds it back to the human body, thereby canceling the common-mode voltage, reducing the displacement current, and effectively suppressing the common-mode interference. The STM32F103 chip is used as the microcontroller unit (MCU) of the monitoring system. The chip has high performance (72 MHz operating frequency, single-cycle multiplication instructions, and hardware partition instruction), low power consumption (0.19 mW/MHz), and rich peripherals. The technical information of the proposed hardware system is shown in Table 1. The sampling rate of the acquisition system is Fs = 500 Hz, and the data are recorded with a resolution of 24 bits.

## 3. Algorithm for Signal Analysis

**Figure 2:**
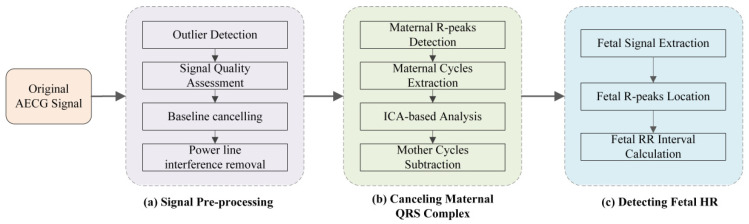
The structure block diagram of the fetal ECG extraction and fQRS location using the algorithm proposed in this work. (**a**) Signal Pre-processing. (**b**) Canceling Maternal QRS Complex. (**c**) Detecting Fetal HR.

The framework of the proposed approach undertaken in this work includes three steps: (1) Signal pre-processing. The AECG signals are processed for removing these invalid values by spline interpolation method; signal quality assessment (SQA) for AECG to get a better-quality signal based on SampEn; signal noise canceling (SNC) for AECG by eliminating power line interference, baseline drift, and impulsive artifacts based on Notch filter, Butterworth filter, and median filter, respectively. (2) A source separation algorithm is applied to MECG subtraction for the FECG signal extraction. (3) Fetal QRS complexes detection algorithm is performed on the filtered residual signals containing FECG signal. The structure block diagram of fQRS location using the algorithm proposed in this paper is presented in Figure 2.

Furthermore, the other dataset employed in this work is from PhysioNet/Computing in Cardiology Challenge 2013 Database (PCDB) [34]. The PCDB consists of 447 recordings from five different databases. Seventy-five AECG recordings are included in training set A. Each recording contains four channels of AECG signals, and they are 60 s long and sampled at 1 kHz with 16-bit resolution. Reference annotations are generated by experts referring to direct FECG signals, obtained from fetal scalp electrodes.

### 3.1. Signal Preprocessing

#### 3.1.1. Signal Quality Assessment

AECG signal may contain invalid values because of human body movement. Therefore, the signal needs to be preprocessed to remove these invalid values. The cubic spline interpolation is applied to eliminate invalid values in this work. It is a challenging task for fQRS complexes detection from the original AECG signals due to a variety of noise. Signal quality assessment (SQA) plays a crucial role in physiological signal processing and inaccurate characteristic estimation [35]. The sample entropy (SampEn) has been a vital tool in SQA for ECG signal processing for the selection of the waveform, with reference to the article from Liu [36]. The calculation process of SampEn is summarized below:

For RR segment *x*(*i*) (1 ≤ *i* ≤ *N*), given the parameters *m* and *r*, firstly form the vector sequences $X_{i}^{m}$:

(1) $$X_{i}^{m}=\left\{x(i),x(i+1),\ldots ,x(i+m-1)\right\},\;1\leq i\leq N-m$$

where the vector $X_{i}^{m}$ represents *m* consecutive *x*(*i*) values. The definition of the distance between $X_{i}^{m}$ and $X_{j}^{m}$ based on the maximum absolute difference is below:

(2) $$d_{i,j}^{m}=d\left[X_{i}^{m},X_{j}^{m}\right]=\underset{0<k<m-1}{\max}\left|x(i+k)-x(j+k)\right|$$

For each $X_{i}^{m}$, express $B_{i}^{m}(r)$ as (*N* − *m*)^−1^ times the number of $X_{j}^{m}$ (1 ≤ *j* ≤ *N* − *m*) that meets $d_{i,j}^{m}$ ≤ *r*. Similarly, denote $A_{i}^{m}(r)$ as (*N* − *m*)^−1^ times the number of $X_{j}^{m+1}$ that meets $d_{i,j}^{m}$ ≤ *r* for all 1 ≤ *i* ≤ *N* − *m*.

The definition of SampEn is as follows:

(3) $$\mathrm{SampEn}(m,r,N)=-\ln(\sum_{i=1}^{N-m}A_{i}^{m}(r)/\sum_{i=1}^{N-m}B_{i}^{m}(r))$$

where *m* is the embedding dimension, *r* represents the tolerance threshold, and *N* is the time-series length.

At first, the AECG signal in each channel is divided into six non-overlapping segments (10 s for each segment), and the average of their corresponding SampEn values is returned as the SampEn result of the current channel. Then, the signal quality is evaluated by comparing the SampEn value in each channel with a constant threshold, which is set to 1.5 for AECG recordings. The average SampEn value is greater than 1.5 for the channels that are regarded as poor quality and are excluded. The signal of the channel whose mean sampEn is less than 1.5 is regarded as good quality signal and is reserved. If less than two channels are of good quality, the two channels with the penultimate and the smallest SampEn values are reserved.

#### 3.1.2. Signal Noise Canceling

The original AECG signals usually contain power line interference, baseline drift, and impulsive artifacts. Power line interference includes a sinusoidal component at a frequency of around 50 Hz, which significantly affects signal quality. Baseline drift is mainly caused by human movement and breathing, and it is manifested in lots of AECG signals. These noises have a harmful influence on the analysis and processing of the signal. The notch filter is utilized to remove the power line interference in this work [37]. The combination of Butterworth filter and median filter is applied to eliminate baseline drift and impulsive artifacts. The power line interference, baseline drift, and impulsive artifacts of the AECG are mostly eliminated after the signal noise-canceling step.

### 3.2. Maternal QRS Detection and Mother Cycles Subtraction

#### 3.2.1. Maternal QRS Detection

The primary step for the R complex location is preprocessing, which consists of wavelet transform analysis, absolute value transformation, and low-pass filtering step. The preprocessing here is to convert the original ECG signal into a signal composed of a single pattern of peaks. Compared with the original signal, the preprocessed signal is more convenient for detection. After performing the preprocessing steps on the signal, the adaptive dual threshold (ADT) algorithm is employed to locate the R-wave peak of the signal [38,39]. The ADT approach sets a high threshold (thr_H) and a low threshold (thr_L) to extract the R wave. If thr_L is smaller than lim_L, then thr_L = lim_L thr_L = lim_L. If *P* is greater than thr_L, and *P* is smaller than thr_H, then thr_L = 0.45 × P and thr_H can be described as:

(4) thr_H=thr_L+|P−mean(P_prior)|/2

If *P* is greater than thr_L, then thr_L and thr_H can be expressed as:

(5) thr_L=0.3×mean(P_prior)

(6) thr_H=0.7×mean(P_prior)

where *P* is the amplitude of the current peak value, *P_prior* represents the sum of the amplitude of the stored 10 peaks before the current peak value, and lim_L and lim_H are the two empirical constants obtained after multiple trials, representing the lower limits of the two threshold changes, respectively.

#### 3.2.2. Mother Cycles Subtraction

It is well known that maternal ECG (MECG) is a major component of the AECG signal and masks fetal ECG signals. Thus, it is necessary to effectively cancel the MECG signal. Blind source separation (BSS) is described as solving the problem of separating or estimating the source waveform from the sensor array without knowing the characteristics of the transmission channel. The BSS method assumes that the source signals are statistically independent, so the independent components of the original signal can be obtained from the multi-channel AECG signal. The independent component analysis (ICA) approach is one of the commonly used methods in BSS, and it is also one of the most promising methods in current blind source separation. The ICA method is capable of eliminating the high-order statistical correlation in the observed signal by achieving the maximum value of the objective function of a certain contrast function and realizing blind source separation [40].

The correct application of ICA requires meeting the following conditions: (1) The signal source is statistically independent; (2) The number of measurement signals is greater than the number of signal sources. The mother’s myocardium is far away from the abdominal leads, and the MECG is the strongest and the most common independent component. Therefore, it can meet the conditions for the ICA application. The ICA approach is exploited to separate the MECG from the other components.

The source signals *S* are expressed as follows:

(7) $$S\left(k\right)=S_{1}\left(k\right),S_{2}\left(k\right),\ldots ,S_{i}\left(k\right)$$

where *S*(*k*) is the independent source signal such as MECG, FECG, and noise, *k* is the time instant, and *i* denotes the number of sources.

The observation signal *X* (the AECG recording) obtained from the sensor nodes is:

(8) $$x\left(k\right)=x_{1}\left(k\right),x_{2}\left(k\right),\ldots ,x_{j}\left(k\right)$$

where *j* represents the number of AECG recordings utilized.

The observation signal *x* is preprocessed including de-averaging and whitening before using the ICA algorithm. The process of removing the mean value is to subtract the mean value vector *m = E*{*x*} of the signal from the observed signal so that the observed signal becomes a zero-mean variable.

The extraction of the FECG signal is following the equation:

(9) x(k)=AS(k)

where *A* is the mixing matrix, which represents the mixing matrix of *x* after transformations and observations.

Generally, the signal source *S* is acquired by constructing the inverse matrix *W* of the mixing matrix *A*. By constructing the *W* matrix (*W* = *S*^−1^), the estimated independent components *Y* of the source signal can be obtained.

(10) Y=WS=WAS

The residual signal (FECG signal) is obtained by subtracting the maternal cycle template signal from the AECG signal by a combination of the ADT and the ICA algorithm (ADT-ICA-based method).

### 3.3. Fetal QRS Complex Detection

The residual signal may still contain residual noise components, which may potentially affect the estimation and correct identification of fetal QRS complexes. In this work, the wavelet adaptive threshold de-noising method is applied to remove the noise components of the FECG signal. Noise can be effectively removed and a clearer FECG waveform can be gained through this step. Meanwhile, the JADE algorithm is adopted to process the residual signal. Due to the implementation of the JADE algorithm, the FECG signals exhibit enhanced and more pronounced peaks, thus aiding the fetal QRS detection process.

Despite previous steps eliminating noise and artifacts, the power of the fetal complex is still small and mixed with residual noise, resulting in a poor SNR. It weakens the reliability of the criteria for a priori selection of the best channel for fetal ECG detection. The fetal QRS complex location step is performed on the extracted fetal ECG components. Fetal QRS complexes are detected with an adaptive threshold of derivative amplitude, and it is automatically initialized and recursively updated at every new detection [41]. The algorithm searches for the maximum value of the weighted derivative signal. The weights are defined by the trapezoidal window to enhance the samples close to the predicted QRS complex’s location. The fQRS complex detection procedure is applied to all channels. At last, the best estimate of fQRS is selected based on the prior knowledge of typical fetal RR values.

## 4. Experiments and Results

### 4.1. Experiment Design

The three subjects in this work are from the First Affiliated Hospital of Nanjing Medical University. The detailed demographic information of this experiment is presented in Table 2. Additionally, informed consent is obtained from each pregnant woman in this experiment. The subject experimental protocol is approved by the Ethics Committee and the study number is 2020-SRFA-183.

The experiment is designed in a laboratory similar to a home environment to verify the proposed monitoring system can measure fetal signals in different states, including supine, seated, and standing postures. The experiment protocol of this work consists of three steps. At first, pregnant women are asked to maintain in a supine situation for four minutes. The subjects are then asked to sit and monitor AECG signals for another two minutes. Finally, the subjects change to a standing posture and are monitored for an additional two minutes. The pregnant women are around 37 weeks.

### 4.2. Evaluation Performance

In the context of NI-FECG extraction, a matching window of 50 ms is applied with each fetal QRS location annotated by experts as a center to evaluate the error of fQRS complexes detection (i.e., fQRS within a ±50 ms window) [42]. If the detected fQRS location is in the matching window, it illustrates that the detected fQRS is the correct value. The performance of this study is evaluated in terms of sensitivity (*Se*), positive predictive accuracy (*PPV*), accuracy (*ACC*), and their harmonic mean (*F*1), following the guideline of the ANSI/AAMI, as defined below:

(11) $$Se=\frac{TP}{TP+FN}$$

(12) $$PPV=\frac{TP}{TP+FP}$$

(13) $$ACC=\frac{TP}{TP+FP+FN}$$

(14) $$F1=2*\frac{PPV*Se}{PPV+Se}=\frac{2TP}{2TP+FN+FP}$$

where *TP* is the number of True Positive that match the fetal QRS marked by experts (correctly detected fetal QRS complexes), *FP* stands for False Positive (wrongly detected fetal QRS complexes), and *FN* represents False Negative (missed detected fetal QRS complexes). 

Bland–Altman graph is a simple and intuitive way of illustrating the consistency of data. The basic idea of the Bland–Altman method is to calculate the mean difference between the two sets of measurement results and take the 95% agreement limit as the mean difference (1.96 SD). Therefore, the Bland–Altman plot could be employed to further evaluate the accuracy value of the detected fetal heart rate. The 95% limit is expected to be chosen to test the difference between the estimated fetal heart rate values of the proposed method and the reference annotations.

### 4.3. Results

**Figure 3:**
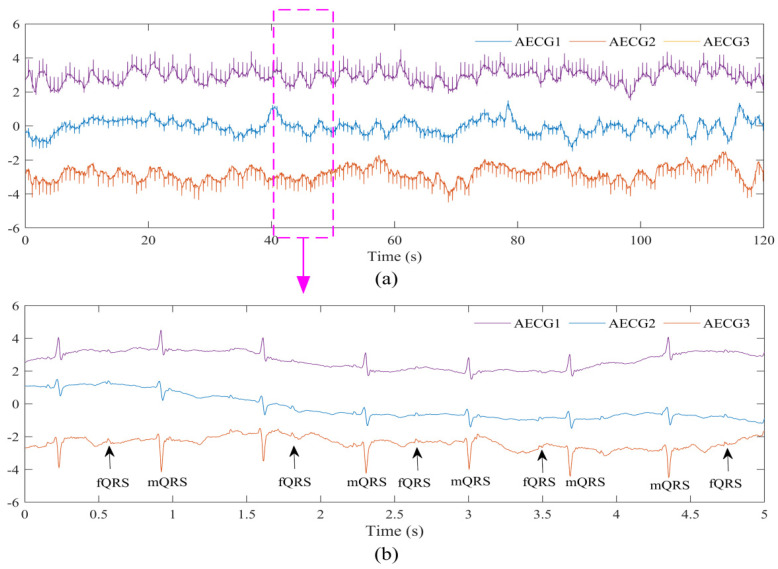
The collected AECG signal of subject A, who simulated supine posture (DS-database). (**a**) AECG signal with 120 s length interval. (**b**) AECG signal with 5 s length interval. (**b**) is a zoom of (**a**).

Our method has been implemented in Matlab 2019b (MathWorks Inc., Natick, MA, USA). The dataset collected in this experiment (DS-database) consists of eight AECG recordings (r01–r12) from three different subjects. Each recording contains 2-min long three AECG signal channels for a total of 3309 fQRS waves. The reference annotations are generated by the experts. The collected AECG signal of subject A, who simulated supine posture, is shown in Figure 3. It can be seen that the quality of the AECG signal is good, and a clear fQRS can be observed.

**Figure 4:**
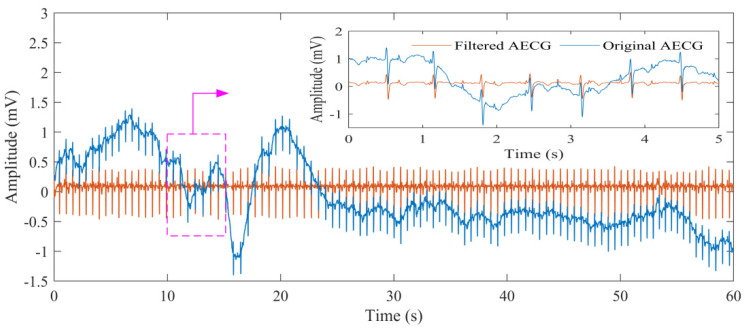
The original AECG recording and the filtered AECG after SNC (DS—database).

**Figure 5:**
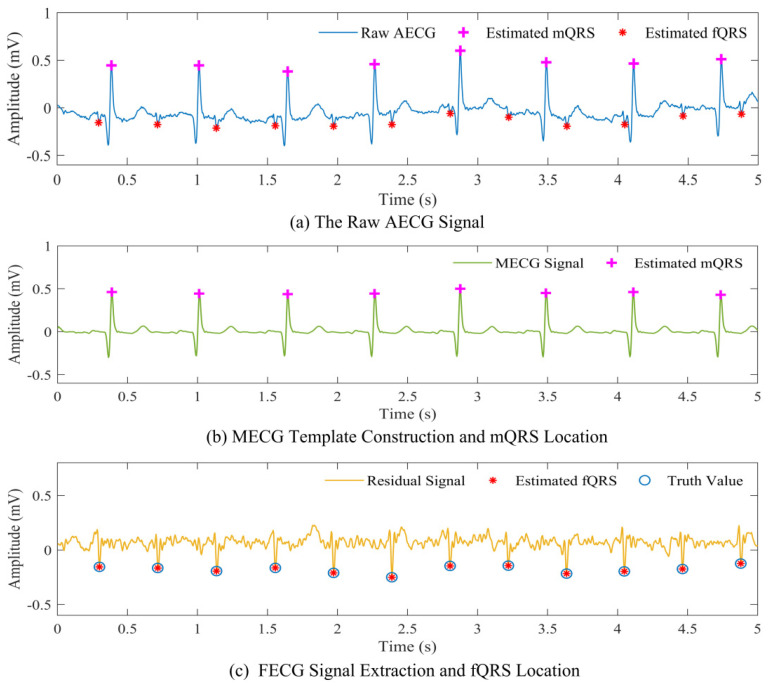
Part of the result of the extraction process of MECG signal, mQRS location, fetal ECG signal, and fQRS location (DS-database). (**a**) An example result of mQRS and fQRS estimation on the raw AECG signal. The ‘+’ represents the mQRS location position, and the ‘*’ represents the fQRS location position. (**b**) A MECG template signal is extracted and mQRS location on the extracted MECG signal. The ‘+’ represents the mQRS location position. (**c**) A FECG template signal is extracted and fQRS location on the extracted MECG signal. The ‘*’ indicates the fQRS location position using the algorithm, and the ‘o’ denotes the truth value annotated by the expert.

Figure 4 shows the original AECG recording and the filtered AECG after SNC. It can be seen that the AECG waveform becomes cleaner after this step. Part of the result of the extraction process of the MECG signal, mQRS location, fetal ECG signal, and fQRS location is shown in Figure 5. It can be seen that the MECG signal and FECG signal are well separated. An example result of mQRS and fQRS estimation using the proposed algorithm on the raw AECG signal is illustrated in Figure 5a. The figure manifests that the mQRS and fQRS wave positions are correctly located, respectively. A visual display of extracted MECG along with the result of mQRS location is exhibited in Figure 5b. In this Figure, we can see the complete MECG signal and correct mQRS position. In addition, the residual signal (i.e., fetal ECG signal) with the location of the estimated fetal R peaks and the truth value annotations of fetal R peaks by the experts is presented in Figure 5c. We are capable of obtaining that the estimated fetal R peak matches the reference annotation.

**Figure 6:**
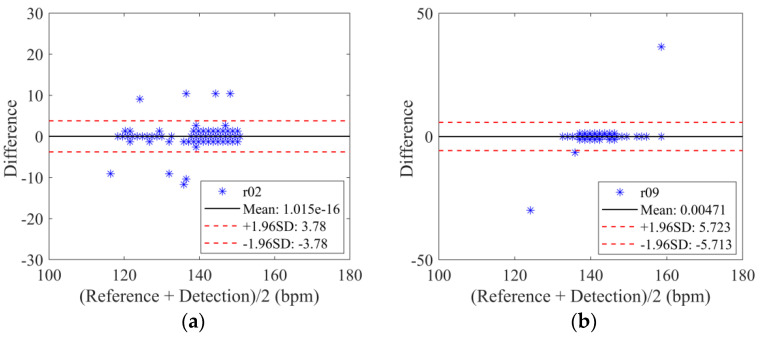
Bland-Altman plot for recording r02 and r09 using the proposed method (DS-database). (**a**) Bland–Altman plot for recording r02. (**b**) Bland-Altman plot for recording r09.

The Bland–Altman statistical analysis method for the estimated fetal heart rate values of the proposed method and the reference annotations (recording r02 and r09) is displayed in Figure 6. The results show that most of the values lie within the 95% interval for the recording of r02 and r09. Performance metrics of the fQRS detection using this method on the DS-database are summarized in Table 3. We can obtain the correct number of fQRS wave detections, and the number of errors detected for each AECG recording. The average diagnostic *Se*, *PPV*, *ACC*, and *F*1 score are 99.62%, 97.90%, 97.40%, and 98.66%, respectively.

**Figure 7:**
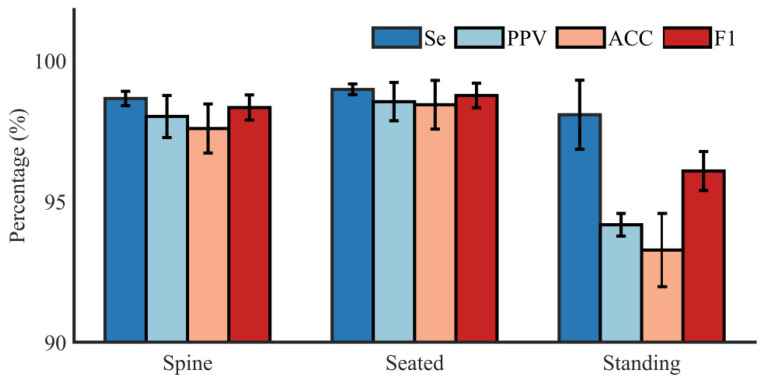
Intuitive results of *Se*, *PPV*, *ACC*, and *F*1 score of all the subjects in supine, seated, and standing postures (DS-database).

Intuitive results of *Se*, *PPV*, *ACC*, and *F*1 score of all the subjects in supine, seated, and standing postures are shown in Figure 7. It demonstrates that the average *Se*, *PPV*, *ACC*, and *F*1 score in spine posture are 99.52%, 98.82%, 98.35%, and 99.17% with a standard deviation of 0.28%, 0.82%, 0.96%, and 0.49%, respectively. When the subjects changed their posture from supine to seated, the mean *Se*, *PPV*, *ACC*, and *F*1 score are 99.88%, 99.4%, 99.28%, and 99.64% with a standard deviation of 0.21%, 0.75%, 0.95%, and 0.48%, respectively. In the standing position, the mean *Se*, *PPV*, *ACC*, and *F*1 score are 98.89%, 94.59%, 93.60%, and 96.69% with a standard deviation of 1.35%, 0.44%, 1.43%, and 0.76%, respectively. Compared to the results in the standing position, the results in supine and seated postures perform better. The results in the seated positions performed slightly better than the results in the supine position.

## 5. Discussion

In this work, we develop a portable, home-based FECG monitoring system that can be used to monitor pregnant women and fetuses’ health. The combination of ADT and the ICA algorithm is implemented to realize the FECG waveform and fetal heart rate extraction. The Monica AN24 and Avalon fetal monitoring systems are capable of acquiring FECG signals. However, those two devices are not the most convenient solutions for home-based monitoring owing to the demand for professional skills and knowledge to utilize the acquisition device [9]. Compared to the home-based monitoring solutions that were previously mentioned, our FECG monitoring system has done signal acquisition verification and fetal heart rate analysis in different postures (supine, seated, and standing).

It is worth mentioning that we can see the FECG signal clearly from the displayed AECG signal in Figure 3. The result in Figure 5 proves that the extracted FECG signals from the collected AECG signals are relatively clear and usable, and thus can serve as a valuable source of information for fetal health state monitoring. It needs to be emphasized that the quality of the AECG signal is of paramount importance for reliable morphological analysis of pregnant women and fetuses. Therefore, it also puts forward higher requirements for the acquisition module of the monitoring system. Furthermore, to satisfy the reliability of the subsequent signal analysis, SampEn is applied to assess the quality of the AECG signal. Subsequently, we extracted the FECG signal from the preprocessed AECG data in the database and collaborated with experts to annotate the fQRS, ensuring the accuracy of the data annotation.

Table 3 demonstrates the diagnostic *PPV*, *ACC*, and *F*1 score of r04 (standing) are 94.55%, 92.20%, and 95.94%, respectively. The diagnostic *PPV*, *ACC*, and *F*1 score of r08 (standing) are 94.18%, 93.54%, and 96.66%, respectively. In addition, the diagnostic *PPV*, *ACC*, and *F*1 score of r12 (standing) are 95.05%, 95.05%, and 97.46%, respectively. The signal fetal R wave detection results in the standing posture are worse than those in the supine and seated states, which might be related to the relatively poor signal quality in the standing posture. In the standing state, the fetus has more space and more time for movement, resulting in poor signal quality.

Table 4 summarizes the performance of *PPV*, *ACC*, and *F*1 score on the PCDB and DS-database between our work and other popular approaches. As shown in Table 4, the result of this work outperformed the approaches of CNN, TS, AF, and FUSE method. According to statistical analysis, the proposed approach truly detects 3291 (*TP*) fetal QRS complexes and wrongly detects 18 (*FP*) fetal QRS complexes for all AECG recordings from the DS-database. Diagnostic Se, PPV, ACC, and F1 score are 99.46%, 97.89%, 95.86%, and 98.67% from the DS-database, respectively. Furthermore, the proposed approach truly detects 9210 (*TP*) fetal QRS complexes and wrongly detects 372 (*FP*) fetal QRS complexes for all AECG recordings from the PCDB database. Diagnostic *PPV*, *ACC*, and *F*1 score are 96.12%, 96.20%, 92.67%, and 96.16% from the PCDB database, respectively. The average *PPV*, *ACC*, and *F*1 score from this work is slightly higher than the fetal QRS detection result of the TS algorithm and FUSE method. Meanwhile, the experimental result of this work is significantly higher than the fetal QRS location result of the CNN and AF approach. It also proves that the proposed approach will greatly improve the accuracy of detection of the fetal QRS complexes.

The adaptive filter method is theoretically more suitable, but the result closely depends on the presence of a signal in chest leads and the setting of the adaptive filter. Additionally, optimal filter settings may vary with the position of the fetus in the uterus and the mother’s gestational week, etc. The non-adaptive approach provides the superiority of using only abdominal electrodes without thoracic electrodes. In our future study, we focus on utilizing a combination of adaptive and non-adaptive methods for fetal ECG signal analysis and R wave extraction. It is worth knowing that the hybrid method should be able to accurately extract fetal signals and perform better in the analysis of the fetal signal.

The present work has only tested short-term recordings in three postures (supine, seated, and standing), and consistent signal quality over longer monitoring periods has not been experimentally demonstrated. Moreover, the number of subjects is small, and a power analysis conducted is not performed.

## 6. Conclusions

In this study, we develop a portable, home-based FECG monitoring application system that can be applied to health monitoring in different postures (supine, seated, and standing) of pregnant women. The result reveals that the quality of the AECG signal in supine and seated postures performs better than that of standing posture. The combination of ADT and the ICA algorithm is incorporated for FECG signal extraction with JADE algorithm for enhanced quality of the FECG, which can provide an accurate and reliable FHR estimation. The fetal health monitoring system contributes in terms of medical resources and physician time. The system is suitable for pregnant women and has certain application prospects.

In the future, the data collection of more participants will be carried out to verify the practicability of the wearable fetal monitoring system in a 24-h or long-term monitoring system.

## Figures and Tables

**Table 1:** The technical information of the proposed hardware system.

Parameter	Value
Sampling rate	500 Hz
Input voltage	−185–185 mV
ADC resolution	24 bits
Gain	24
Power supply	3.7 V 1000 mAh
Input referred noise	2.4 μVpp
Input impedance	1000 MΩ
Size	55 mm × 55 mm

**Table 2:** The detailed demographic information of the subjects in this experiment.

Statistical Information	Age (Years)	Height (cm)	Weight (kg)
Average	29	159	65
Standard Deviation	1.8	1.2	5.4

**Table 3:** Performance metrics of the fQRS detection using this method.

Subject	Recording	Position	*TP*	*FP*	*FN*	*Se* (%)	*PPV* (%)	*ACC* (%)	*F*1 (%)
A	r01	Supine	282	3	1	99.65	98.95	98.60	99.30
	r02	Supine	281	0	0	100	100	100	100
	r03	Seated	280	1	0	100	99.64	99.64	99.82
	r04	Standing	260	15	7	97.38	94.55	92.20	95.94
B	r05	Supine	267	4	1	99.63	98.53	98.16	99.07
	r06	Supine	284	8	2	99.30	97.26	96.60	98.27
	r07	Seated	274	0	0	100	100	100	100
	r08	Standing	275	17	2	99.28	94.18	93.54	96.66
C	r09	Supine	275	2	2	99.28	99.28	98.57	99.28
	r10	Supine	271	3	2	99.27	98.91	98.19	99.09
	r11	Seated	273	4	1	99.64	98.56	98.20	99.09
	r12	Standing	269	14	0	100	95.05	95.05	97.46

**Table 4:** Comparison of performance metrics for fetal QRS detection of different subjects in different methods.

Database	Approach	*Se* (%)	*PPV* (%)	*ACC* (%)	*F*1 (%)
PCDB	CNN [24]	76.00	82.00	-	78.00
	TS [25]	-	-	-	93.90
	FUSE method [30]	95.90	96.00	-	0.9600
	This work	96.12	96.20	92.67	96.16
DS-database	TS [25]	98.37	96.59	93.40	97.47
	AF [26]	90.18	92.87	86.69	91.51
	This work	99.46	97.89	95.86	98.67

## Data Availability

Not applicable.
